# From Microfiche to Meta‐Analysis: How Literature Reviewing Changed

**DOI:** 10.1111/jan.70590

**Published:** 2026-04-06

**Authors:** Helen Aveyard

**Affiliations:** ^1^ Oxford Brookes University Oxford UK

When I started my own doctoral study in 1996, there was no easy way to search for research, and there was no clear expectation that the background research that underpinned a doctoral study would be comprehensively examined. This was partly due to expectation of the day and partly due to the available technology: which of course were interwoven. Access to research was possible using technology such as microfiche‐ microscope to read minutarised print journals. A comprehensive review was possible, but these were far less common and generally a ‘review’ resembled an essay from which it was not possible to tell if the included papers had been cherry picked from a wider body of evidence. Thirty years on, expectations have shifted profoundly. The literature review is a key component of any doctoral, postdoctoral and even at master's and undergraduate level research we now expect that a review is undertaken systematically. In addition, a standalone review in which evidence about a specific question is summarized, is a core component of academic life. Literature reviewing has moved beyond the narrow question of effectiveness and opened up space for examining meaning, experience, context and process, areas central to nursing inquiry. So, what has happened to lead to these changes?

## 1 Influence of the Cochrane Collaboration

Firstly, in the early 1990s, the Cochrane Collaboration and Campbell Collaboration were established that championed an explicit, systematic approach to doing a review about the effectiveness of a treatment or intervention. Often referred to as a systematic review with meta‐analysis, the Cochrane Collaboration approach is widely accepted as the gold standard for doing a review of the effectiveness of interventions or care procedures (Higgins et al. 2026)—in other words, to find out does the intervention actually work or provide benefit to the patient? If so, at what cost, both financially and in consideration of side effects experienced by the patient. These reviews generally incorporate the findings from randomized controlled trials which, on their own, might be too small to be conclusive but when combined with the findings of other trials, provide more robust evidence. Reviews undertaken in this way provide clear evidence of the usefulness of an intervention. These systematic reviews have revolutionized health and social care, removing the guess work and estimation from the recommendation of what effective care and treatment might comprise.

## 2 Development of Other Methods for Doing a Review

Secondly, the development of methods for doing a systematic review with meta‐analysis of randomized trials was a trigger for the development of systematic methods for doing literature reviews of other study types to answer other kinds of questions. For example, Noblet and Hare published their text for the synthesis of qualitative studies in 1988. This was followed by other approaches to reviewing qualitative research. At around this time, Whittemore and Knafl (2005) published their well‐known approach to the synthesis of both qualitative and quantitative research and non‐research papers, using an integrative review method. These approaches reflect just a few methods that are available for those doing a literature review. I believe that the impact and influence of the Cochrane and Campbell Collaboration reviews was a driving force behind the drive for more systematic approaches to reviews of different types of study, moving them out of the category of ‘essay’. As a consequence, academic study for those doing a literature review for questions that fall outside the ‘does it work?’ category can now be undertaken transparently and systematically.

## 3 Commitment to Evidence Based Practice

Thirdly, I also believe that greater acknowledgement of the benefits of doing literature reviews using an explicit logic and method enhances our commitment to evidence‐based practice. Evidence based practice is a term which has been re‐named over the years as evidence informed care, evidence‐based nursing and so on, but the central message is unchanged: Care should be delivered which is based or informed by evidence in addition to established practice, professional judgement and patient preference. In many areas, though not all, we are fortunate to have a large body of evidence which can inform practice. Given that it is not practical for busy practitioners to read every academic paper relating to their practice, and any one academic paper provides just one piece of evidence, without a review of literature, much research can go un‐noticed. Thus, well conducted literature reviews are vital in developing an evidence‐based approach to care.

These factors have led to both the drive and technology to undertake literature reviews. The development of electronic academic databases has enabled advanced methods for searching and has been accompanied by the role of information specialists within academic libraries. There is software to facilitate the organization and analysis of research. Literature reviews provide an undoubted benefit for our profession. But looking forward to the next 50 years, what are the challenges?

## 4 Challenges for the Next Generation of Reviews

### 4.1 Proliferation Without Consolidation

The first challenge is that the methods for doing a review have proliferated in recent years. Grant and Booth identified 14 different types in 2009 whereas Aveyard and Bradbury‐Jones identified at least 35 in 2019 in a review of trends in the publication of reviews. Estimates of the number of review types vary, but the proliferation of different types of reviews is evident. Furthermore, some refer to a named review type and others' names are constructed by the reviewers themselves. To complicate the field further, Aveyard and Bradbury‐Jones (2019) identified that distinct methods are not always adhered to—there can be drift away from the prescribed method—hence presenting a confusing picture. In addition, the term ‘systematic’ is often applied to a review name, presumably to indicate rigour, which can lead to confusion as it is not yet clear whether the term ‘systematic’ is one that is (or should be) synonymous with a literature review with meta‐analysis or whether it should be used as an umbrella term to describe any review which has been undertaken in a systematic way. There is currently no clear consensus on this, but the inconsistent use of the term in different contexts is potentially confusing for readers who might wonder how a ‘systematic integrative review’ differs from an ‘integrative review.’ To counter this challenge, Aveyard and Bradbury‐Jones (2019) called for clarity and consolidation in the methods used by researchers when doing a literature review. They advocate adherence to a published method for doing a literature review to ensure that authors build on and consolidate existing methods rather than develop additional approaches which might further complicate the field.

### 4.2 Volume, Quality and Trust

The second challenge is to acknowledge that there is an ever increasing amount of evidence, often accessible though an increasing variety of online platforms. The importance of being able to recognize good quality evidence has never been more important; whether this is a review, a piece of research or argument. The need to be discerning about the quality of evidence is a fundamental skill that needs to be introduced in our schools and continually followed up as students commence nurse education and beyond. The proliferation in the amount of literature that is available to all within the profession is a concern to us all. Whilst we value the synthesis of research and other evidence in a review, we must be mindful of the need to recognize well conducted research from other weaker forms of evidence and ensure that appropriate and authentic research is included in the reviews undertaken. Theis includes the need to distinguish reliable evidence from fake or fabricated evidence. This is a key skill which students and registered professionals must develop and one about which I have written about (e.g., Aveyard and Waite (2025)).

### 4.3 Navigating Reporting Guidelines Across Diverse Review Types

The third challenge is the appropriate use of reporting guidelines which academic journals often require their authors to adhere to. These outline which aspects of a review should be reported to enhance transparency and demonstrate rigour and confidence in the published paper. Reporting guidelines are currently available on the EQUATOR network website. Reporting guidelines which are relevant for those doing a literature review include PRISMA‐P (2015 statement) for systematic reviews with meta‐analysis and the ENTREQ guidelines (Tong et al. 2012) for qualitative reviews. However, due to the many different types of reviews, there is not an associated publishing guideline for every type of literature review. Therefore, it is not currently possible to require that all those doing a literature review adhere to an existing reporting guideline as this risks shoehorning the reporting of a variety of methods inappropriately into guidelines that may not be applicable. Therefore, the universal recourse to a publishing guideline is not always possible and those who use reporting guidelines should be cognizant of this.

### 4.4 Artificial Intelligence and Scholarly Judgement

Related to this, a fourth challenge is artificial intelligence (AI). Whether or not AI poses an existential threat to the jobs of systematic reviewers is sometimes discussed. In my view, the current answer to this question is a firm no. AI does not currently have access to the research included in subject‐specific academic databases such as CINAHL and thus any review created by AI will be incomplete. AI can be used to undertake discrete steps in the review process; for example, it can do a follow‐up check to see if any additional papers can be identified following a database search. It can be used to undertake an initial appraisal of papers; however, this is not necessarily done to the specificity needed for the requirements of a review, so the results of any appraisal undertaken in this way need to be studied carefully. There is no doubt that some of the steps undertaken in a review might be replaced by AI in the future, but this seems to me to be some way ahead. Furthermore, I argue that the process of doing a literature review is a valuable learning process and one through which the research gains many skills that we would not want to lose to AI. However, all authors need to be aware of fake papers, fake references and indeed fake reviews.

### 4.5 The Risk of Proceduralism

The fifth challenge is the potential for a formulaic or recipe book approach to doing a review and to lose the depth of understanding that is a key feature of some approaches. Despite the proliferation of names for doing a literature review, there are many common features: a focused research question, a planned search strategy, data extraction and critical appraisal and an appropriate level of synthesis so that new findings come from the review, depending on the review type. It is at the point of synthesis that a clear divergence between different types of reviews can often be found. Those doing a systematic review with meta‐analysis will undertake a synthesis of the statistics in the included papers, whereas those doing an analysis involving the description or interpretation of the findings of different papers will present their results narratively. It has been observed that the level of synthesis on many papers is low, and findings are often presented as a simple list (Thorne 2017), thus losing the level of interpretation that is the value of a systematic review.

## 5 Concluding Reflections

At the *Journal of Advanced Nursing*, we receive and welcome a great many literature reviews for potential publication and we are committed to publishing a selection of those which have been authentically undertaken. We rely on authors to ensure that the included papers meet the inclusion criteria of the review and that all sources are rigorously checked for authenticity. The review should adhere to an established, referenced method and be accompanied by a clear and transparent account of the method, following the structure of a relevant reporting guideline. With the proliferation of papers, including fake references, we recognize that this is a complex area; it is one that is more important than ever to get right. With our authors, peer reviewers, editors and readers we look forward to engaging with in further debate so that we move the science of doing a literature review forwards.

Looking ahead, one of the most pressing tasks for nursing scholars is not simply to refine review methods further, but to exercise scholarly judgment and stewardship in how they are used. The next generation of nurse researchers will inherit an evidence landscape that is vast, uneven in quality and increasingly shaped by automation and artificial intelligence. In this context, the ability to think critically about what evidence matters, how it is synthesized and whose voices are amplified will be as important as technical competence in any specific review method. Literature reviews should remain sites of intellectual engagement and interpretation, rather than procedural exercises undertaken for compliance alone. As the *Journal of Advanced Nursing* enters its next 50 years of scholarship, there is an opportunity; even a responsibility, to ensure that literature reviewing continues to support the development of a rich, inclusive and practice‐relevant nursing knowledge base. Ongoing dialogue and debate about how best to review literature will be essential if the science and craft of literature reviewing is to continue to evolve over the next 50 years.

## Funding

The author has nothing to report.

## Conflicts of Interest

The author declares no conflicts of interest.

## Data Availability

The author has nothing to report.
